# Donor Skeletal Muscle Quality Affects Graft Mortality After Living Donor Liver Transplantation- A Single Center, Retrospective Study

**DOI:** 10.3389/ti.2022.10723

**Published:** 2022-12-09

**Authors:** Takahiro Tomiyama, Noboru Harada, Takeo Toshima, Yuki Nakayama, Katsuya Toshida, Akinari Morinaga, Yukiko Kosai-Fujimoto, Takahiro Tomino, Takeshi Kurihara, Kazuki Takeishi, Yoshihiro Nagao, Kazutoyo Morita, Shinji Itoh, Tomoharu Yoshizumi

**Affiliations:** Department of Surgery and Sciences, Graduate School of Medical Sciences, Kyushu University, Fukuoka, Japan

**Keywords:** sarcopenia, intramuscular adipose tissue content, small-for-graft size syndrome, donor muscle status, graft quality

## Abstract

The recipient muscle status is closely associated with postoperative poor survival in recipients of living donor liver transplantation (LDLT). However, it is uncertain whether LDLT donor muscle quality and quantity affect graft quality. Hence, we analyzed the correlation between donor muscle status and graft function. We measured the skeletal muscle mass index (SMI) and intramuscular adipose tissue content (IMAC) of 380 LDLT donors. We examined the correlation between donor SMI or IMAC and graft mortality, the occurrence rates of small-for-size graft (SFSG) syndrome, and 6-month graft survival rates. The donor SMI had no effect on the occurrence of SFSG syndrome and graft survival, while a high IMAC in both male and female donors was significantly correlated with the rate of SFSG syndrome [high vs low: (male donors) 15.8% vs. 2.5%, *p* = 0.0003; (female donors) 12.8% vs. 3.1%, *p* = 0.0234] and 6-month graft survival rates [(male donors) 87.7% vs 95.9%, *p* = 0.02; (female donors) 83.0% vs. 99.0%, *p* < 0.0001]. Multivariate analysis revealed that a high donor IMAC (HR; 5.42, CI; 2.13–13.8, *p* = 0.0004) was an independent risk factor for 6-month graft survival, and the donor IMAC is useful for donor selection for high-risk recipients.

## Graphical Abstract

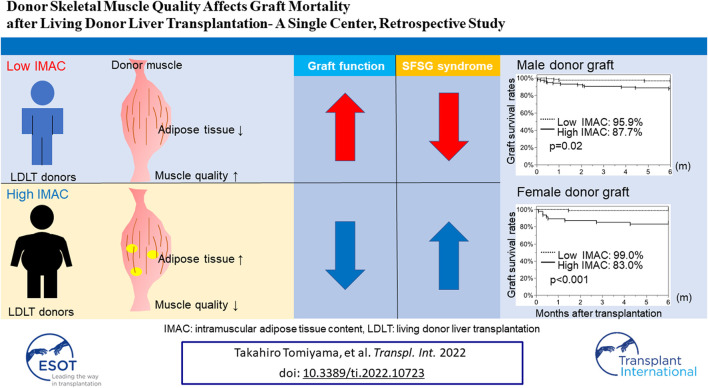

## Introduction

Sarcopenia, defined as an age-dependent decrease in muscle mass and function, is reportedly an independent risk factor of poor survival in the presence of several diseases (1 –5). In the field of liver transplantation, preoperative recipient sarcopenia is reportedly correlated with increasing sepsis and mortality rates in recipients after living donor liver transplantation (LDLT) (6). In addition, the transplant recipient preoperative skeletal muscle mass-to-visceral fat area ratio, visceral adiposity, low muscularity, and high intramuscular adipose tissue content (IMAC) are closely associated with high postoperative recipient mortality following LDLT (7, 8). These findings indicate that low quantity and quality of muscle in the recipient preoperatively closely correlate with postoperative mortality in LDLT recipients.

However, these correlations with liver transplantation and muscle quality and quantity are not surprising because the liver is strongly affected by muscle tissue (9). Skeletal muscle tissue secretes a hormone, called myokine, which regulates muscle metabolism, increases insulin sensitivity, and influences adipose tissue mass and fat deposition in the liver (10,11). On the other hand, adipose tissue can release hormones, called adipokines, which regulate lipid metabolism, decrease insulin sensitivity, and influence fibrogenesis in the liver (10, 12). Thus, skeletal muscle is closely involved in determining the liver condition.

The effect of skeletal muscle in LDLT donors has not been fully examined. LDLT donors are healthy and lack severe comorbidities. Preoperative blood tests are performed to confirm that there are no abnormalities. In addition, donor liver steatosis and cold ischemic time are reportedly graft quality markers in deceased donor liver transplantation (DDLT) (13, 14). If a donor has mild obesity or fatty liver in LDLT, dietary restriction and exercise are implemented, and LDLT is performed after complete improvement of obesity and fatty liver. In LDLT, the cold ischemic time is very short and much less likely to be affected compared to DDLT. The population of LDLT donors is quite homogeneous compared to that of DDLT donors. However, there is diversity in LDLT donor body shape and muscle mass. In LDLT, exercise and diet improve the health of the donor (15), and regular exercise reduces intrahepatic adiposity, increases β-oxidation of fatty acids, and induces hepato-protective autophagy (16). Hence, donor muscularity may reflect the health status of the liver and the condition of the graft, and it may be useful to base the decision of donor selection in LDLT on donor muscularity.

In the present study, the pretransplant donor skeletal muscle mass index (SMI) and IMAC were retrospectively evaluated, and the impact of the SMI and IMAC on graft survival was assessed.

## Methods

### Patients

The study protocol was approved by the Institutional Review Board of the Kyushu University Hospital, approval number 2019–354. This study was conducted according to the Declaration of Helsinki of 1996. Written informed consent was obtained from all patients before LDLT. In total, 380 adult patients (age >17 years) underwent LDLT at Kyushu University Hospital, Japan, between January 2007 and March 2018. Recipients who could be followed for at least 6 months after LDLT were included. If an LDLT donor had mild obesity or fatty liver, dietary restriction and exercise were implemented, and LDLT was performed after complete improvement of obesity and fatty liver. In our cohort, only two donors underwent weight loss before the donation.

### Image Analysis

**FIGURE 1:**
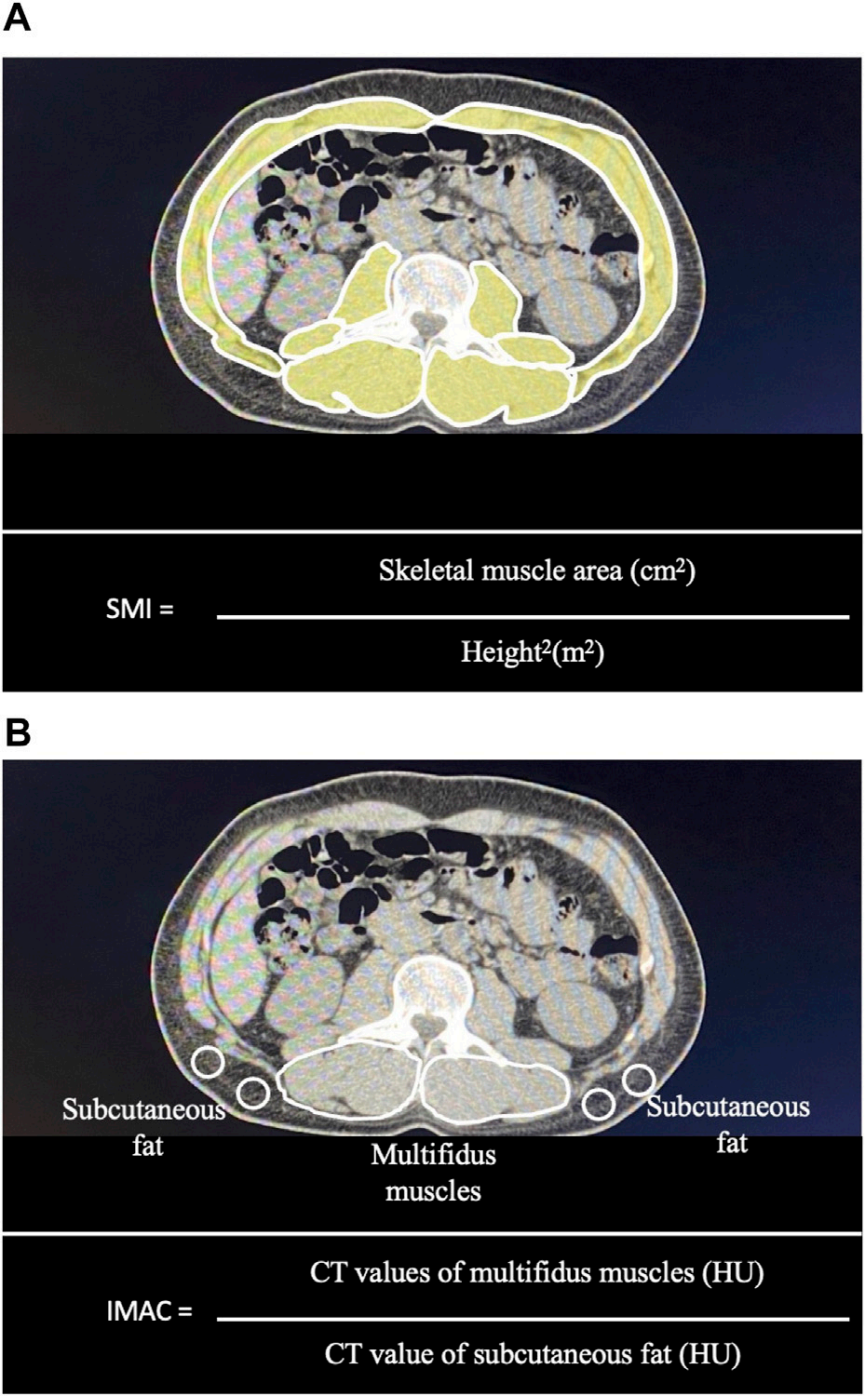
Measurement of the skeletal muscle mass index (SMI) and intramuscular adipose tissue content (IMAC) using a cross-sectional CT image obtained at the third lumbar vertebral level: **(A)** Skeletal muscle areas were measured by manual tracing. Skeletal muscle areas were normalized with patient height in m^2^ and expressed as the SMI. **(B)** The preoperative mean CT value for the right and left multifidus muscles (in Hounsfield units, HU) was divided by the mean CT value for four points of subcutaneous fat and expressed as the IMAC.

Computed tomography (CT) scanning was performed within 1 month preoperatively. The SMI and IMAC were calculated as previously reported (5, 17). Briefly, the skeletal muscle area and IMAC were calculated using cross-sectional CT images obtained at the third lumbar vertebral level. Skeletal muscle areas were measured by manual tracing and normalized with patient height in m^2^ and expressed as the SMI (Figure 1A). The preoperative mean CT value for the right and left multifidus muscles (in Hounsfield units, HU) was divided by the mean CT value for four points of subcutaneous fat and expressed as the IMAC (Figure 1B). A higher IMAC indicates a larger amount of adipose tissue in the skeletal muscle and, therefore, muscle that is of poorer quality.

### Selection Criteria

The selection criteria for the recipients and donors have been previously described (18, 19).

The selection criteria for LDLT for patients without hepatocellular carcinoma (HCC) were as follows: 1) no other potentially curative modality available and 2) no other organ failure present. There was no limitation on recipient age. The selection criteria for LDLT for patients with HCC were as follows: 1) no other potentially curative modality available, 2) no extrahepatic metastasis, and 3) no major vascular invasion. The Model for End-Stage Liver Disease (MELD) score was calculated using a formula reported by Kamath et al. (20).

Donors were selected from among candidates who had volunteered for the procedure (18). They were required to be within three degrees of consanguinity or the spouse of the recipient and to be between 20 and 65 years of age. For donors not within three degrees of consanguinity with the recipient, individual approval was obtained from the Ethic Committee of Kyushu University Hospital. Good Samaritan donation was not used. The standard liver volume of recipients was calculated according to the formula of Urata (21). Three-dimensional CT was performed for volumetric analysis and delineation of vascular anatomy. Decisions regarding graft type were based on the preoperatively predicted graft volume/recipient standard liver volume (GV/SLV) ratio. Left lobe + caudate lobe grafts were basically used when the preoperatively predicted GV/SLV ratio was ≥35%, but relatively small grafts, such as those with a GV/SLV between 30% and 35%, were selected when the donor was younger than 30 years of age (18). When the GV/SLV ratio of the left lobe + caudate lobe graft was <35% and remnant liver volume after right lobectomy was ≥35%, a right lobe graft was used. A posterior segment graft was considered when the donor’s vascular anatomy was suitable for this purpose (22).

### Surgical Technique

The graft procurement technique and recipient surgery have been previously described (23). Splenectomy was performed using a vessel sealing system (Ligasure; Covidien Japan, Tokyo, Japan) and automatic suturing device (Endo GIA, Covidien Japan or Powered ECHELON, ETHICON, New Brunswick, NJ, United States) as described previously (19, 24).

### Postoperative Management

The perioperative management of recipients, including the immunosuppression regimens, have been described previously (18, 19, 25). Briefly, immunosuppression was initiated using a protocol based on either tacrolimus (Prograf: Astellas Pharma, Tokyo, Japan) or cyclosporine A (Neoral; Novartis Pharma K.K, Tokyo, Japan) with mycophenolate mofetil (CellCept; Pfizer, New York, America) and steroids. The target trough concentration for tacrolimus was set at 10 ng/ml for 3 months after LDLT, followed by 5–10 ng/ml. The target trough concentration for cyclosporine A was set at 250 ng/ml for 3 months after LDLT, followed by 150–200 ng/ml. Methylprednisolone was initiated on the day of the LDLT, after which the dose was tapered, and prednisolone was sustained 7 days after the LDLT. Prednisolone treatment was tapered and discontinued 6 months after LDTL. Mycophenolate mofetil was used, beginning with 2 or 3 g on the day after LDLT; the dose was tapered and discontinued 6 months after LDLT. The trough concentration of mycophenolate mofetil was not measured.

Portal, hepatic arterial, and hepatic venous flows were assessed using Doppler ultrasonography twice per day until postoperative-day (POD) 7 and once per day thereafter during the first admission. For recipients with simultaneous splenectomy and LDLT, portal vein thrombosis prevention was not routinely performed. When the platelet count increased to 500,000/ml or higher during the follow-up period, 100 mg of aspirin was administered, which was discontinued when the platelet count decreased below 500,000/ml.

The abdominal drain was removed when the daily ascites volume became lower than 500 ml.

### Endpoints

The primary endpoint was 6-month graft survival. If there was a significant difference in the first endpoint, we also examined laboratory data and the amount of abdominal drainage as secondary endpoints. Six-month graft loss was defined as recipient death or re-transplantation within 6 months.

### Parameters Analyzed

#### Data Analysis

Categorical variables are presented as numbers and percentages and all patient background information was compared using the Pearson’s chi-square test. Based on their distributions, continuous variables are presented as the mean with 95% confidence interval, and they were compared using the *t*-test.

Graft survival data were analyzed using the Kaplan–Meier method and compared using the log-rank test. Continuous variables were compared using the *t*-test and categorical variables were compared using the chi-squared (?^2^) test. Any variable in the univariate analysis was identified as significant at *p* < 0.05, or variables at *p* < 0.2 were considered candidates for the multivariate Cox analysis. The results are shown as hazard ratios (HRs) with 95% confidence intervals (CIs). A value of *p* < 0.05 was considered to indicate statistical significance. All statistical data were generated using JMP Pro 15 (SAS Institute, Cary, NC, United States).

## Results

### Measurement of Donor SMI and IMAC and Examination of the Cutoff Value

**FIGURE 2:**
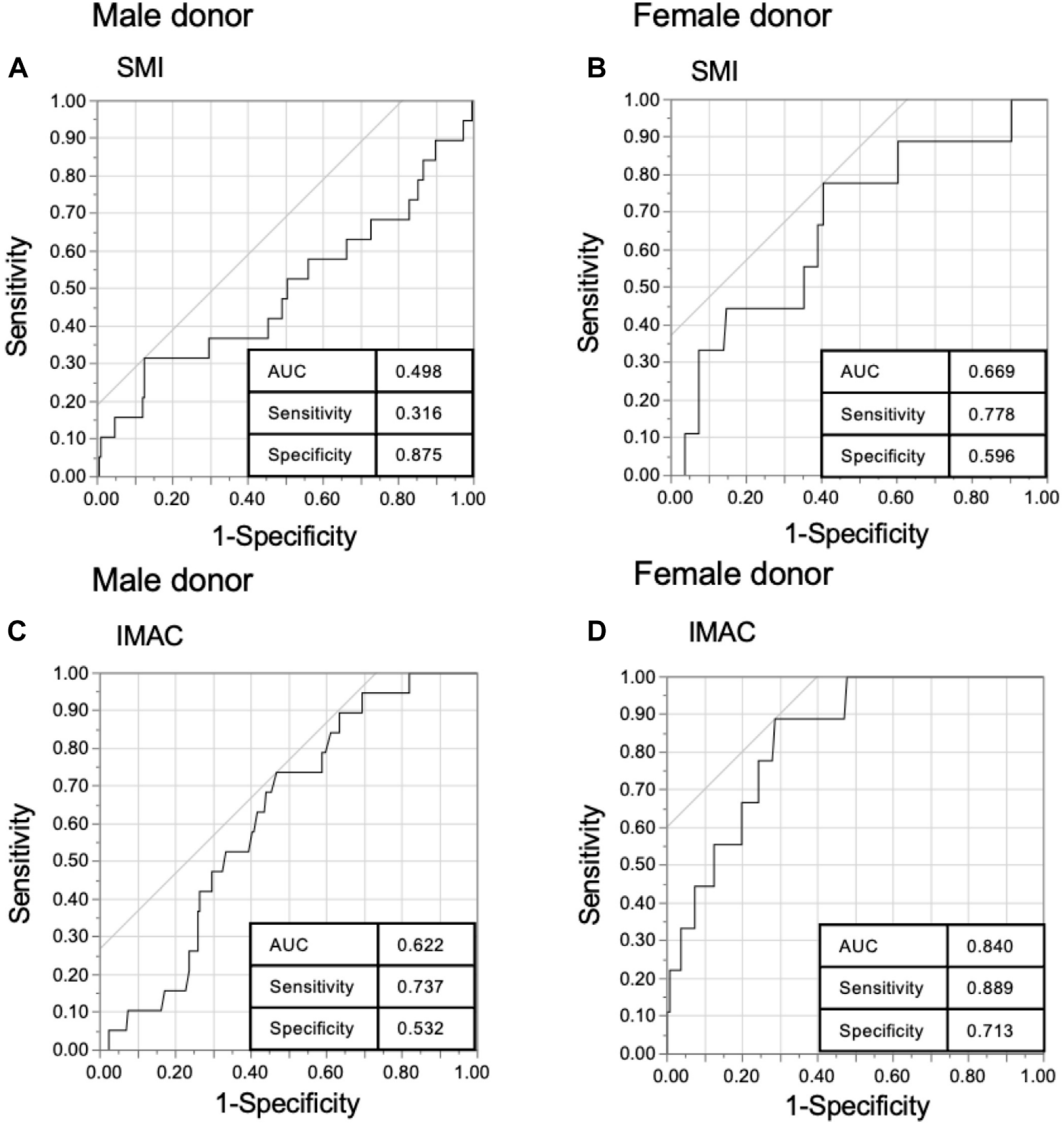
Receiver operating characteristics (ROC) curve of the donor muscle mass index (SMI) and intramuscular adipose tissue content (IMAC). ROC of **(A)** male donor SMI, **(B)** female donor SMI, **(C)** male donor IMAC, and **(D)** female donor IMAC with 6-month graft survival in living donor liver transplantation.

There was a significant difference in donor SMI and IMAC by sex [male donor (*n* = 235) vs. female donor (*n* = 145); mean SMI: 50.1 vs. 39.4, *p* < 0.0001, mean IMAC: −0.557 vs. −0.507, *p* < 0.0001]. Thus, we separated data from male and female donors for further analysis. The optimal cutoff values for predicting primary 6-month graft loss were derived from receiver operating characteristic curves, with SMI cutoff values for men and women of 57.0 and 37.5 (sensitivity 31.6% and 77.8%, respectively; specificity 87.5% and 59.6%, respectively), and IMAC cut-off values for men and women of −0.553 and −0.473, respectively (sensitivity 73.7% and 88.9%, respectively; specificity 53.2% and 71.3%, respectively) (Figures 2A–D).

### Correlation of Preoperative Donor Muscle Condition With Patient Characteristics

As shown in Table 1, depicting the male donor SMI analysis, there were significant differences in the rates of right lobe grafts and sepsis after transplantation between the low-SMI and high-SMI groups (low-SMI group vs. high-SMI group; right lobe rate: 58.3% vs. 76.7%, *p* = 0.0248, sepsis rate: 6.3% vs. 16.3%, *p* = 0.0292). Table 2 shows the patient characteristics in female donors. Cold ischemic time was significantly longer in the low-SMI group than in the high-SMI group (157 min vs. 113 min, *p* = 0.0013), but there was no significant difference in recipient postoperative parameters. Table 3 shows the patient characteristics for the male donor IMAC analysis. In the high-IMAC group, the rates of right lobe graft and GV/SLV were lower (38.2% vs. 40.8%, *p* = 0.0283) and intraoperative recipient blood loss was higher (3.90L vs. 3.79L, *p* = 0.0362) than in the low-IMAC group. Regarding recipient postoperative parameters, the admission period was longer (29 vs. 24 days, *p* = 0.0492) and the rates of SFSG syndrome (15.8% vs. 2.5%, *p* = 0.0004) and graft failure (12.3% vs. 4.13%, *p* = 0.0220) were higher in the high-IMAC than in the low-IMAC group. Table 4 shows the patient characteristics for the female donor IMAC analysis. The rate of ABO incompatibility was lower (8.51% vs. 22.5%, *p* = 0.0406) and the MELD score was higher (19 vs. 16, *p* = 0.0005) in the high-IMAC group than in the low-IMAC group. Regarding postoperative parameters, the rates of sepsis, SFSG syndrome, and graft failure were higher in the high donor IMAC group than in the low donor IMAC group (sepsis; 14.9% vs. 2.41%, *p* = 0.0027; SFSG syndrome; 12.8% vs. 3.1%, *p* = 0.0234; graft failure within 6-month; 17.0% vs. 1.02%). In both the male and female analyses, there was no significant difference in the cause of graft loss.

**TABLE 1:** Difference in patient characteristic between male high SMI group and low SMI group.

Variables	Male donor SMI		*p*-value
	High (*n* = 43)	Low (*n* = 192)	
Preoperative donor variables			
Age (years)	33 (20–62)	36 (20–63)	0.0636
Graft (right lobe)	10 (23.3%)	80 (41.7%)	0.0248
Actual GV/SLV (%)	40.3 (23.2–73.1)	39.8 (22.6–70.1)	0.7135
Actual GRWR (%)	0.787 (0.430–1.35)	0.770 (0.397–1.42)	0.7616
ABO (Incompatible)	5 (11.6%)	32 (16.7%)	0.4122
Recipient preoperative variables			
Age (years)	55 (19–76)	57 (20–74)	0.3485
Sex (male)	15 (34.9%)	78 (40.6%)	0.4865
Primary diagnosis			
Hepatocellular disease	30 (69.8%)	130 (67.7%)	
Cholestatic disease	20 (20.9%)	32 (16.7%)	
Others	4 (9.30%)	30 (15.6%)	
HBsAb (yes)	9 (20.9%)	50 (26.0%)	0.5070
HCVAb (yes)	18 (41.9%)	70 (36.5%)	0.5082
HCC (yes)	19 (44.2%)	76 (39.6%)	0.5783
Body mass index (kg/m^2^)	24.1 (15.8–32.0)	23.7 (14.9–35.6)	0.9234
ICU or hospital statement (yes)	14 (32.6%)	67 (34.9%)	0.7706
DM (yes)	10 (23.3%)	38 (19.8%)	0.6105
MELD	14 (4–29)	15 (4–54)	0.0760
Splenectomy (yes)	32 (74.4%)	163 (84.9%)	0.0985
Pre-transplant WBC count (x10^3^/μL)	4.06 (1.46–15.7)	4.06 (0.39–20.6)	0.5546
Pre-transplant Platelet count (x10^4^/μL)	7.40 (2.60–44.6)	6.85 (0.90–30.2)	0.0710
Intraoperative parameters			
Recipient operation time (h)	12.2 (8.33–21.6)	12.1 (7.55–24.8)	0.7576
Recipient blood loss (L)	3.80 (0.15–68.3)	3.84 (0.12–220)	0.7711
Cold ischemic time (min)	81.5 (42–210)	92 (35–376)	0.0550
Warm ischemic time (min)	38 (28–125)	41 (25–119)	0.6401
PVP before the end of operation	16 (9–25)	16 (6–30%)	0.6908
Recipient postoperative parameter			
Admission period (day)	25 (13–78)	25 (1–145)	0.4970
Sepsis	7 (16.3%)	12 (6.3%)	0.0292
SFSG syndrome	3 (7.0%)	18 (9.4%)	0.6183
Acute cellular or humoral rejection	7 (16.3%)	18 (9.38%)	0.1844
Graft failure within 6 months	5 (11.6%)	14 (7.29%)	0.3458
Cause of graft loss within 6 months			
Liver failure	2 (40.0%)	8 (57.1%)	
Sepsis	1 (20.0%)	4 (28.6%)	
Others	2 (20.0%)	2 (14.3%)	0.4805

Data are presented as median (range) or n (%).

DM, diabetes mellitus; GRWR, graft recipient weight ratio; GV/SLV, graft volume/recipient standard liver volume ratio; HBsAg, hepatitis B surface antigen, HCC, hepatocellular carcinoma; HCVAb, hepatitis C virus antibody; HCC, hepatocellular carcinoma; ICU, intensive care unit; MELD, Model for End-Stage Liver Disease; PVP, portal vein pressure; SFSG, small-for-size graft.

**TABLE 2:** Difference in patient characteristic between female high SMI group and low SMI group.

Variables	Female donor SMI		*p*-value
	High (*n* = 89)	Low (*n* = 56)	
Preoperative donor variables			
Age (years)	38 (21–64)	39 (21–62)	0.2019
Graft (right lobe)	61 (68.5%)	45 (80.4%)	0.1182
Actual GV/SLV (%)	42.5 (26.9–63.0)	40.6 (28.9–56.4)	0.2386
Actual GRWR (%)	0.782 (0.524–1.21)	0.755 (0.509–1.19)	0.3869
ABO (Incompatible)	17 (19.1%)	9 (16.7%)	0.6433
Recipient preoperative variables			
Age (years)	56 (17–73)	71 (21–71)	0.4245
Sex (male)	39 (43.8%)	33 (58.9%)	0.0765
Primary diagnosis			
Hepatocellular disease	62 (70.0%)	36 (64.3%)	0.3052
Cholestatic disease	20 (22.5%)	11 (19.6%)	
Others	7 (7.87%)	9 (16.1%)	
HBsAb (yes)	14 (15.7%)	10 (17.9%)	0.7372
HCVAb (yes)	28 (31.5%)	15 (26.8%)	0.5484
HCC (yes)	24 (27.0%)	17 (30.4%)	0.6589
Body mass index (kg/m^2^)	23.3 (17.0–32.9)	23.3 (17.2–29.0)	0.4330
ICU or hospital statement (yes)	25 (28.4%)	21 (37.5%)	0.2540
DM (yes)	10 (11.2%)	8 (14.3%)	0.5876
MELD	16 (5–44)	17 (5–45)	0.2161
Splenectomy (yes)	78 (87.6%)	48 (85.7%)	0.7379
Pre-transplant WBC count (x10^3^/μL)	3.99 (1.04–15.9)	4.20 (1.17–15.8)	0.9446
Pre-transplant Platelet count (x10^4^/μL)	7.00 (1.2–36.2)	6.25 (1.7–34.8)	0.7092
Intraoperative parameters			
Recipient operation time (h)	12.6 (8.10–18.0)	12.5 (8.47–20.7)	0.2898
Recipient blood loss (L)	3.70 (0.58–26.4)	5.62 (0.20–50.4)	0.0770
Cold ischemic time (min)	113 (39–261)	157 (50–367)	0.0013
Warm ischemic time (min)	41 (25–103)	44.5 (22–83)	0.4413
PVP before the end of operation	15 (7–25)	14.5 (9–22)	0.5789
Recipient postoperative parameter			
Admission period (day)	27 (9–172)	29 (3–80)	0.2890
Sepsis	5 (5.62%)	4 (7.14%)	0.7110
SFSG syndrome	6 (6.7%)	3 (5.4%)	0.7366
Acute cellular or humoral rejection	7 (7.87%)	5 (8.93%)	0.8210
Graft failure within 6 months	7 (7.87%)	2 (3.57%)	0.2968
Cause of graft loss within 6 months			
Liver failure	3 (42.9%)	2 (100%)	0.1515
Sepsis	4 (57.1%)	0 (0%)	
Others	0 (0%)	0 (0%)	

Data are presented as median (range) or n (%).

DM, diabetes mellitus; GRWR, graft recipient weight ratio; GV/SLV, graft volume/recipient standard liver volume ratio; HBsAg, hepatitis B surface antigen, HCC, hepatocellular carcinoma; HCVAb, hepatitis C virus antibody; HCC, hepatocellular carcinoma; ICU, intensive care unit; MELD, Model for End-Stage Liver Disease; PVP, portal vein pressure; SFSG, small-for-size graft.

**TABLE 3:** Difference in patient characteristic between male high IMAC group and low IMAC group.

Variables	Male donor IMAC		*p*-value
	Low (*n* = 121)	High (*n* = 114)	
Preoperative donor variables			
Age (years)	34 (20–63)	37 (20–62)	0.1697
Graft (right lobe)	54 (44.6%)	36 (31.6%)	0.0397
Actual GV/SLV (%)	40.8 (26.8–70.1)	38.2 (22.6–73.1)	0.0283
ABO (Incompatible)	0.792 (0.482–1.42)	0.737 (0.397–1.35)	0.0552
Recipient preoperative variables	17 (14.1%)	20 (17.5%)	0.4623
Age (years)	55 (22–74)	57 (19–76)	0.6764
Sex (male)	54 (44.6%)	39 (34.2%)	0.1026
Primary diagnosis			
Hepatocellular disease	86 (71.1%)	74 (64.9%)	0.5522
Cholestatic disease	20 (16.5%)	21 (18.4%)	
Others	15 (12.4%)	19 (16.7%)	
HBsAb (yes)	28 (23.1%)	31 (27.2%)	0.4740
HCVAb (yes)	47 (38.8%)	41 (36.0%)	0.6487
HCC (yes)	53 (43.8%)	42 (36.8%)	0.2773
Body mass index (kg/m^2^)	23.7 (14.9–35.0)	24.1 (15.8–35.6)	0.7142
ICU or hospital statement (yes)	41 (33.9%)	40 (35.1%)	0.8462
DM (yes)	40 (15.8%)	19 (19.6%)	0.3982
MELD	15 (4–54)	15 (4–39)	0.8274
Splenectomy (yes)	102 (84.3%)	93 (81.6%)	0.5794
Pre-transplant WBC count (x10^3^/μL)	4.10 (0.39–16.1)	3.96 (0.96–20.6)	0.5805
Pre-transplant Platelet count (x10^4^/μL)	6.9 (1.8–41.5)	7.0 (0.9–44.6)	0.5164
Intraoperative parameters			
Recipient operation time (h)	12.2 (7.55–23.1)	12.0 (7.57–24.9)	0.3754
Recipient blood loss (L)	3.79 (0.14–29.9)	3.90 (0.12–22.0)	0.0362
Cold ischemic time (min)	88 (35–313)	89 (38–376)	0.9574
Warm ischemic time (min)	41 (26–104)	40.5 (25–125)	0.5239
PVP before the end of operation	15 (6–30)	16 (8–26)	0.1224
Recipient postoperative parameter			
Admission period (day)	24 (9–145)	29 (1–133)	0.0492
Sepsis	7 (5.79%)	12 (10.5%)	0.1827
SFSG syndrome	3 (2.5%)	18 (15.8%)	0.0004
Acute cellular or humoral rejection	13 (10.7%)	12 (10.5%)	0.9569
Graft failure within 6 months	5 (4.13%)	14 (12.3%)	0.0220
Cause of graft loss within 6 months			
Liver failure	3 (60.0%)	7 (50.0%)	
Sepsis	1 (20.0%)	4 (28.6%)	
Others	1 (20.0%)	3 (21.4%)	0.9156

Data are presented as median (range) or n (%).

DM, diabetes mellitus; GRWR, graft recipient weight ratio; GV/SLV, graft volume/recipient standard liver volume ratio; HBsAg, hepatitis B surface antigen, HCC, hepatocellular carcinoma; HCVAb, hepatitis C virus antibody; HCC, hepatocellular carcinoma; ICU, intensive care unit; MELD, Model for End-Stage Liver Disease; PVP, portal vein pressure; SFSG, small-for-size graft.

**TABLE 4:** Difference in patient characteristic between female high IMAC group and low IMAC group.

Variables	Female donor IMAC		*p*-value
	Low (*n* = 98)	High (*n* = 47)	
Preoperative donor variables			
Age (years)	37 (21–60)	40 (20–64)	0.2281
Graft (right lobe)	72 (73.5%)	34 (72.3%)	0.8859
Actual GV/SLV (%)	40.7 (26.9–63.0)	42.5 (28.0–54.8)	0.5307
Actual GRWR (%)	0.760 (0.509–1.22)	0.783 (0.563–1.16)	0.6636
ABO (Incompatible)	22 (22.5%)	4 (8.51%)	0.0406
Recipient preoperative variables			
Age (years)	57 (23–71)	58 (17–73)	0.9950
Sex (male)	48 (49.0%)	24 (51.1%)	0.8143
Primary diagnosis			
Hepatocellular disease	63 (64.3%)	35 (74.5%)	
Cholestatic disease	27 (27.6%)	4 (8.51%)	
Others	8 (8.16%)	8 (17.0%)	0.0171
HBsAb (yes)	16 (16.3%)	8 (17.2%)	0.9161
HCVAb (yes)	28 (28.6%)	15 (31.9%)	0.6799
HCC (yes)	25 (25.5%)	16 (34.0%)	0.2856
Body mass index (kg/m^2^)	23.5 (17.0–30.4)	23.0 (17.2–32.9)	0.8357
ICU or hospital statement (yes)	30 (30.9%)	16 (30.0%)	0.7070
DM (yes)	12 (12.2%)	6 (12.7%)	0.9290
MELD	16 (5–36)	19 (9–45)	0.0005
Splenectomy (yes)	84 (85.7%)	42 (89.4%)	0.5424
Pre-transplant WBC count (x10^3^/μL)	4.03 (1.06–15.8)	4.05 (1.04–15.9)	0.0799
Pre-transplant Platelet count (x10^4^/μL)	7.05 (1.2–36.2)	5.70 (1.2–24.3)	0.2080
Intraoperative parameters			
Recipient operation time (h)	12.5 (8.10–20.7)	12.6 (9.33–19.3)	0.2366
Recipient blood loss (L)	3.93 (0.45–50.4)	4.42 (0.20–34.7)	0.7987
Cold ischemic time (min)	129 (39–367)	151 (50–255)	0.5455
Warm ischemic time (min)	43 (23–103)	40 (22–83)	0.2999
PVP before the end of operation	15 (7–25)	15 (7–24)	0.6381
Recipient postoperative parameter			
Admission period (day)	27 (9–172)	30 (3–128)	0.2483
Sepsis	2 (2.04%)	7 (14.9%)	0.0027
SFSG syndrome	3 (3.1%)	6 (12.8%)	0.0234
Acute cellular or humoral rejection	10 (10.2%)	2 (4.26%)	0.2236
Graft failure within 6 months	1 (1.02%)	8 (17.0%)	0.0002
Cause of graft loss within 6 months			
Liver failure	1 (100%)	4 (50%)	
Sepsis	0 (0%)	4 (50%)	
Others	0 (0%)	0 (0%)	0.3428

Data are presented as median (range) or n (%).

DM, diabetes mellitus; GRWR, graft recipient weight ratio; GV/SLV, graft volume/recipient standard liver volume ratio; HBsAg, hepatitis B surface antigen, HCC, hepatocellular carcinoma; HCVAb, hepatitis C virus antibody; HCC, hepatocellular carcinoma; ICU, intensive care unit; MELD, Model for End-Stage Liver Disease; PVP, portal vein pressure; SFSG, small-for-size graft.

**FIGURE 3:**
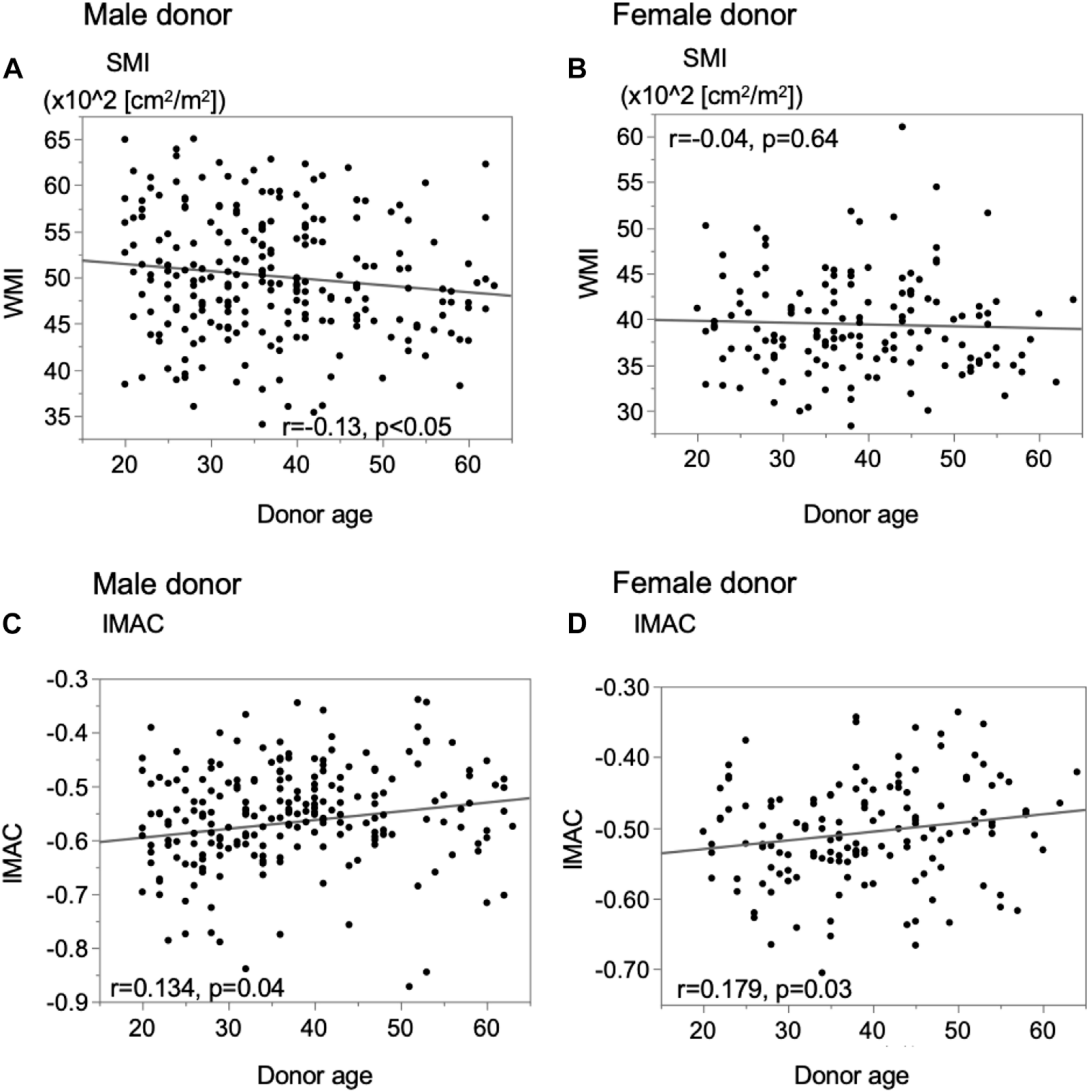
Analysis of the correlation between donor muscle status and donor age. **(A)** Male donor SMI, **(B)** female donor SMI, **(C)** male donor IMAC, and **(D)** female donor IMAC. SMI; muscle mass index, IMAC; intramuscular adipose tissue content.

In male donors, there was a significant negative correlation between preoperative donor SMI and donor age (Figure 3A, *r* = −0.1289, *p* = 0.0484), while for female donors, there was no correlation between the SMI and age (Figure 3B, *r* = −0.0392, *p* = 0.6400). In all donors, there were significant positive correlations between the preoperative donor IMAC and donor age (Figures 3C,D; male; *r* = 0.1340, *p* = 0.0401; female; *r* = 0.1792, *p* = 0.0310).

### Comparison of Graft Function

**FIGURE 4:**
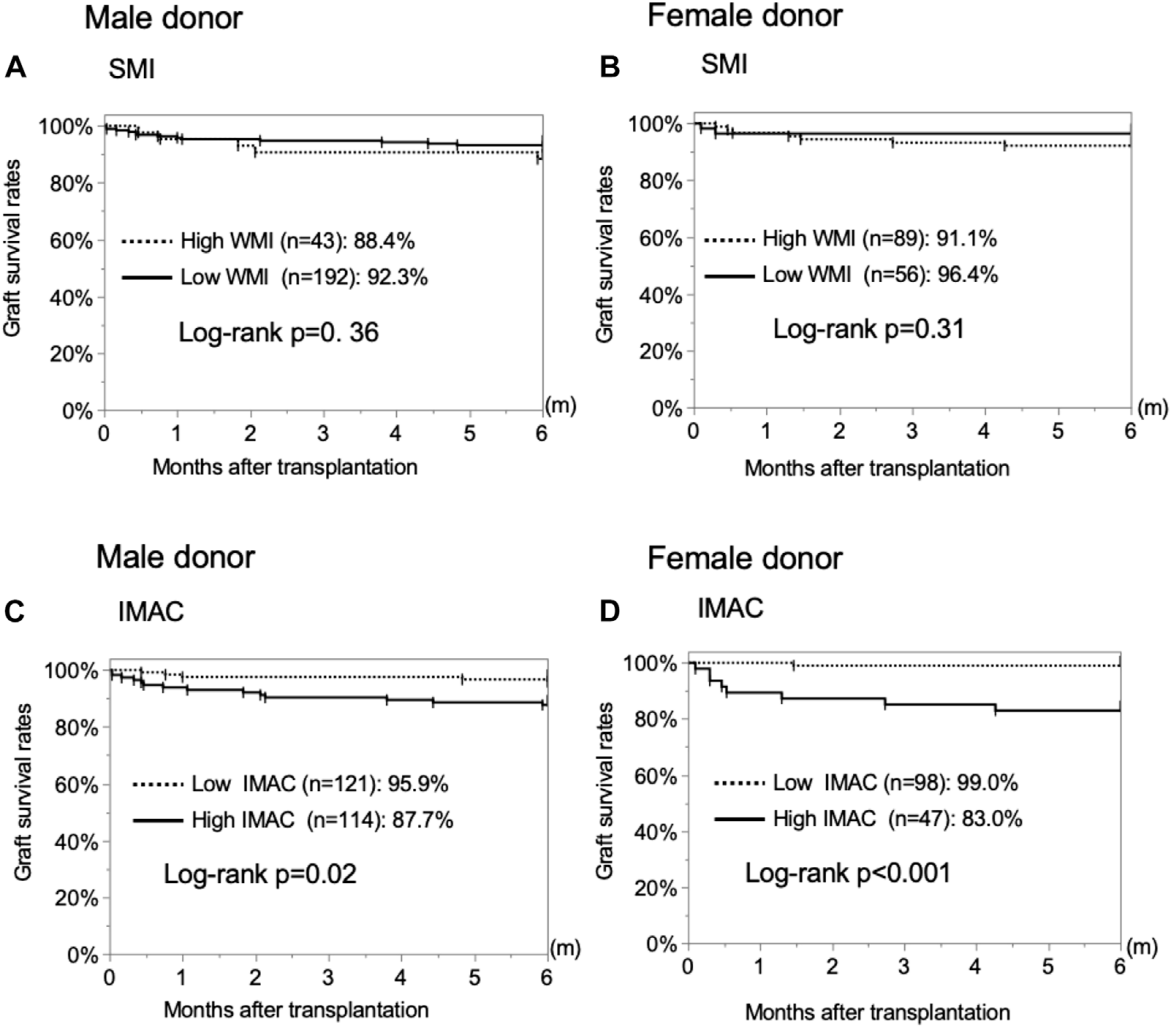
Analysis of the correlation between donor muscle status and 6-month graft survival rates. **(A)** Male donor SMI, **(B)** female donor SMI, **(C)** male donor IMAC, and **(D)** female donor IMAC. SMI, muscle mass index; IMAC, intramuscular adipose tissue content.

The male and female SMI analyses revealed no significant difference in the overall graft survival rates within 6 months after LDLT between the low-SMI group and the high-SMI group (Figures 4A,B; male; 92.3% vs. 88.4%, *p* = 0.3570; female; 96.4% vs. 91.1%, *p* = 0.3128). The male and female IMAC analyses showed that the overall graft survival rates in the high-IMAC group were lower than those in the low-IMAC group (Figures 4C,D; male; 87.7% vs. 95.9%, *p* = 0.0210; female; 83.0% vs. 99.0%, *p* < 0.0001).

**FIGURE 5:**
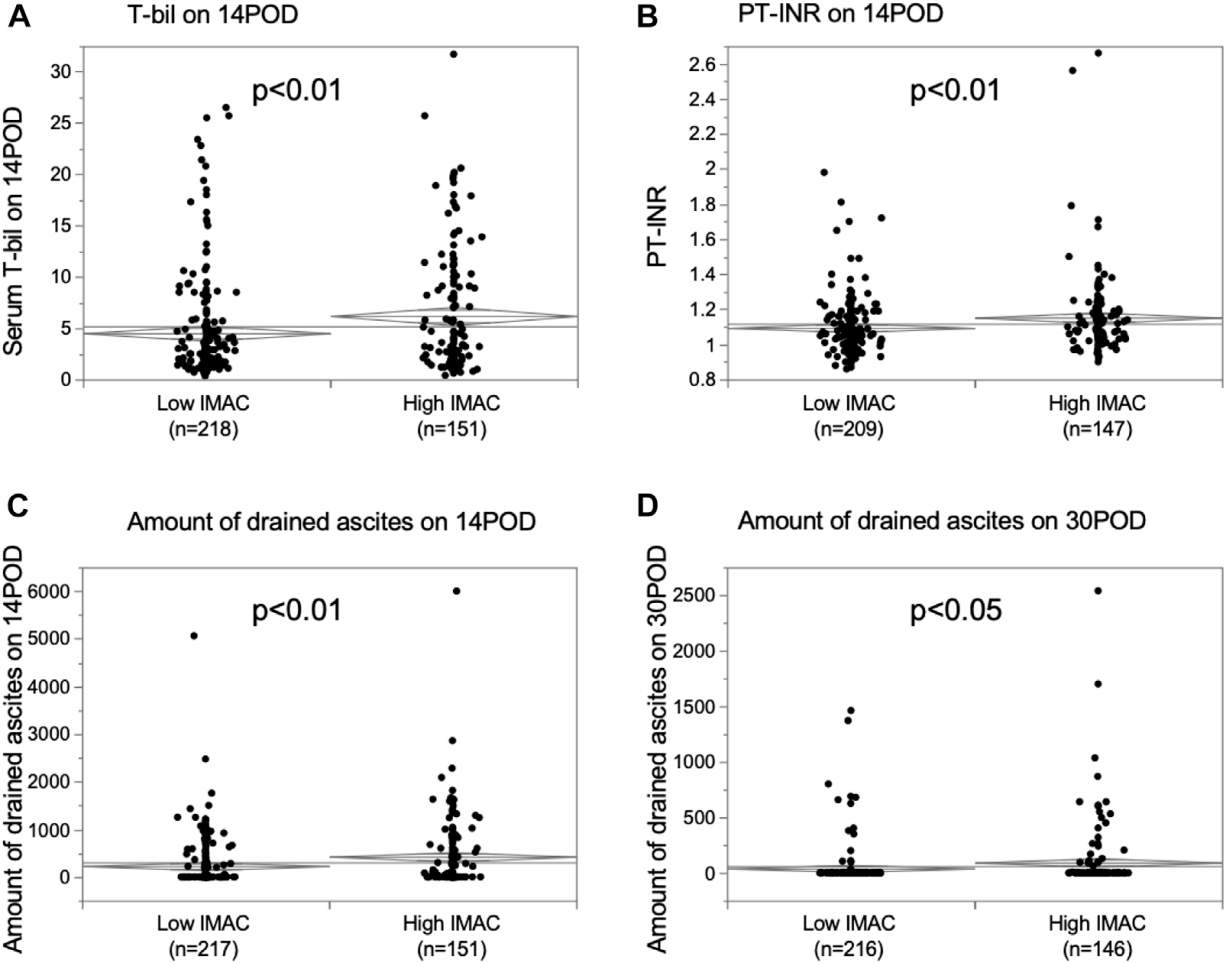
Comparison of graft function in recipients with high IMAC graft and low IMAC graft. **(A)** Serum T-bil on POD 14, **(B)** PT-INR on POD 14, **(C)** amount of ascites on POD 14, **(D)** amount of ascites on POD 30. IMAC, intramuscular adipose tissue content; T-bil, total bilirubin; POD, postoperative day; PT-INR, prothrombin-time international normalized ratio.

The differences in graft function after LDLT between the high- and low-IMAC groups were examined. The serum total-bilirubin (T-bil) level on POD 14, prothrombin-time international normalized ratio (PT-INR) on POD 14, and drained ascites on PODs 14 and 30 were significantly higher in recipients with grafts from high-IMAC donors than from low-IMAC donors (Figures 5A–D; T-bil; 6.2 mg/dl vs. 4.5 mg/dl, *p* = 0.0042; PT-INR: 1.15 vs. 1.10, *p* = 0.0043; ascites on POD 14: 425 ml vs. 228 ml, *p* = 0.0030; ascites on POD 30: 95 ml vs. 41 ml, *p* = 0.0355). We examined liver steatosis in 186 LDLT donors with preserved liver biopsy tissue using hematoxylin and eosin staining to assess the correlation between liver steatosis and IMAC. There were 88 high-IMAC patients and 98 low-IMAC patients. No patient had 5% or higher steatosis in either group. There was no significant difference between the groups in the rate of donors with microvascular steatosis (1%–4%) (high IMAC vs. low IMAC: 13.6% vs. 11.2%, *p* = 0.6179).

### Risk Factors for Poor Graft Survival in Patients Undergoing LDLT

We performed univariate and multivariate cox regression analyses to examine the predictive factors for graft survival within 6 months after LDLT. Table 5 shows the results of the multivariate analysis; high donor IMAC (HR; 5.42, CI; 2.13–13.8, *p* = 0.0004), high MELD score (HR; 2.24, CI; 1.04–4.82, *p* = 0.0384), and absence of splenectomy (HR; 4.94, CI; 2.24–10.9, *p* = 0.0001) were independent risk factors for graft failure within 6 months.

**TABLE 5:** Predictors of graft loss within 6-month.

Variables	Univariate analysis			Multivariate analysis		
	OR	95%CI	*p*-value	OR	95%CI	*p*-value
Donor variables						
WMI						
High (*n* = 132)	1.00	(References)				
Low (*n* = 248)	0.71	0.668–2.99	0.3656			
IMAC						
Low (*n* = 219)	1.00	(References)		1.00	(References)	
High (*n* = 161)	5.13	2.16–13.1	0.0003	5.42	2.13–13.8	0.0004
Sex						
Male (*n* = 235)	1.31	0.592–2.89 (References)	0.5008			
Female (*n* = 145)	1.00					
Age (year)						
<50 (*n* = 318)	1.00	(References)	0.1985	1.00	(References)	0.0514
≥50 (*n* = 62)	1.75	0.745–4.12		2.46	0.994–6.13	
Graft						
Right (*n* = 196)	1.00	(References)	0.1885	1.00	(References)	0.8211
Others (*n* = 184)	1.66	0.779–3.55		1.10	0.383–2.14	
Actual GV/SLV (%) or GRWR (%)						
≥35 and ≥0.7 (*n* = 235)	1.00	(References)	0.1781	1.00	(References)	0.2189
<35 or <0.7 (*n* = 145)	1.66	0.793–3.49		1.66	0.741–3.70	
ABO incompatible						
No (*n* = 317)	1.00	(References)	0.3941			
Yes (*n* = 63)	0.59	0.179–1.97				
Recipient variables						
Sex						
Male (*n* = 165)	1.15	0.546–2.41 (References)	0.7162			
Female (*n* = 215)	1.00					
Age (years)						
<65 (*n* = 309)	1.00	(References)	0.9003			
≥65 (*n* = 71)	0.94	0.357–2.47				
Preoperative DM						
No (*n* = 314)	1.00	(References)	0.5752			
Yes (*n* = 66)	1.29	0.525–3.19				
Hepatocellular disease						
No (*n* = 122)	1.00	(References)	0.3976			
Yes (*n* = 258)	0.72	0.338–1.54				
HCC						
Without HCC (*n* = 244)	1.00	(References)	0.2387			
With HCC (*n* = 136)	0.60	0.254–1.41				
Preoperative hospital treatment						
No (*n* = 252)	1.00	(References)	0.1662	1.00	(References)	
Yes (*n* = 127)	0.53	0.214–1.30		0.59	0.236–1.46	0.2505
MELD score						
≤21 (*n* = 295)	1.00	(References)	0.0114	1.00	(References)	0.0384
>21 (*n* = 85)	2.74	1.30–5.80		2.24	1.04–4.82	
Splenectomy						
With splenectomy (*n* = 321)	1.00	(References)	<0.0001	1.00	(References)	<0.0001
Without splenectomy (*n* = 59)	4.49	2.13–9.51		4.94	2.24–10.9	
Steatosis						
With microvesicular steatosis (*n* = 23)	1.00	(References)	0.7404			
Without steatosis (*n* = 163)		0.09–5.52				

DM, diabetes mellitus; GRWR, graft recipient weight ratio; GV/SLV, graft volume/recipient standard liver volume ratio; HCC, hepatocellular carcinoma; MELD, Model for End-Stage Liver Disease.

### Stratification With Predictive Factors for Graft Survival Within 6 months

**FIGURE 6:**
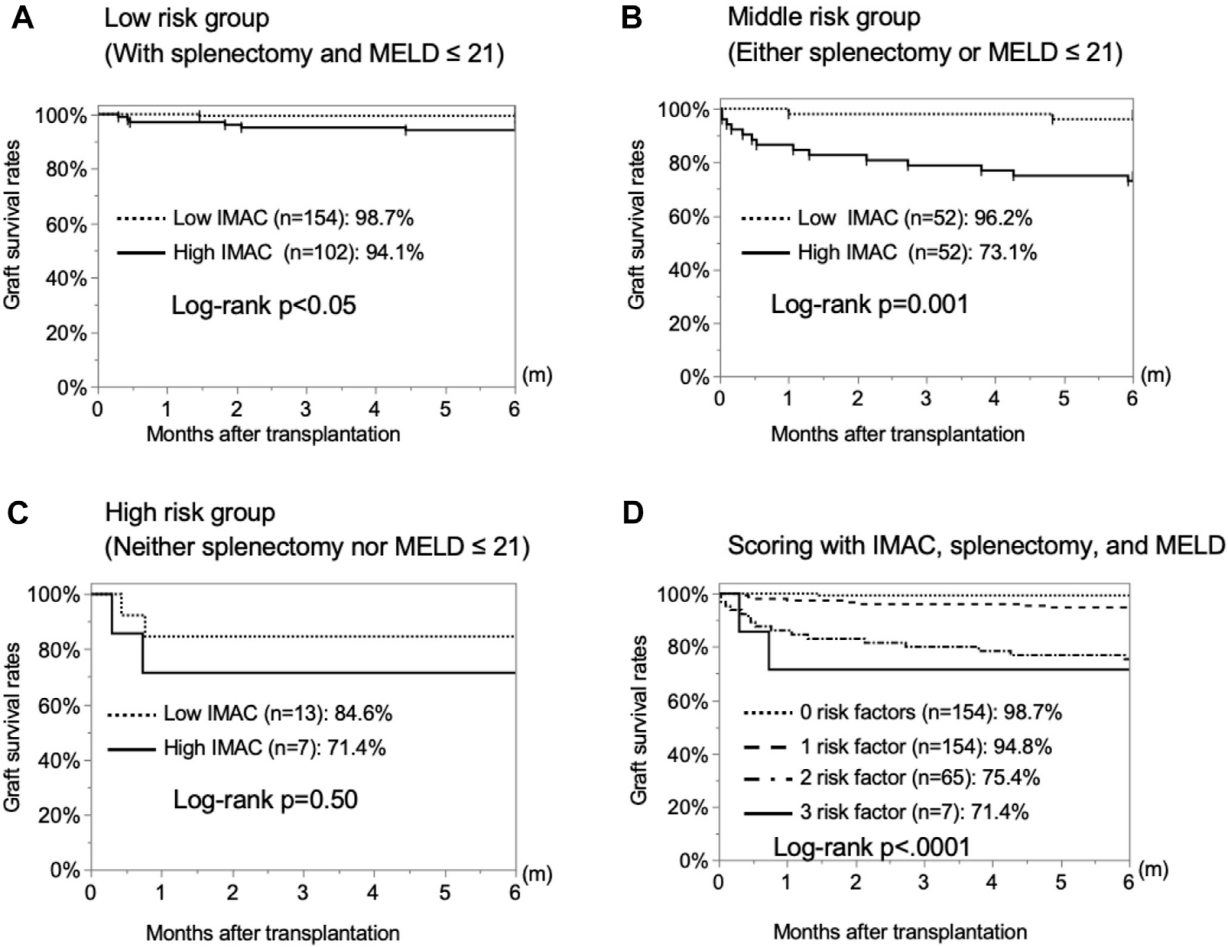
Stratification by risk factors to predict 6-month graft survival rates. The 6-month graft survival rates of recipients with high and low IMAC grafts were examined for each of two risk factors, i.e., absence of splenectomy and high MELD score. **(A)** No risk factors, **(B)** one risk factor, **(C)** two risk factors. **(D)** Stratification for 6-month graft survival rates by the number of present risk factors among the three examined ones.

Next, we examined the significance of donor IMAC for predicting graft survival. We used two risk factors excluding IMAC to stratify the patients into three groups. In the low-risk group, including patients without risk factors, and moderate-risk group, including patients with one risk factor, the graft survival rates in the high-IMAC group were significantly lower than those in the low-IMAC group (low-risk group: 94.1% vs. 98.7%, *p* = 0.0381; moderate-risk group: 73.1% vs. 96.2%, *p* = 0.0010) (Figures 6A,B). However, there was no significant difference between the high- and low-IMAC groups for the high-risk group (84.6% vs. 71.4%, *p* = 0.4995) (Figure 6C). We divided the patients into four groups according to the presence or absence of the three risk factors, and the graft survival rates were stratified according to the number of risk factors (0 risk factors, 98.7%; 1 risk factor, 94.8%; 2 risk factors, 75.4%; 3 risk factors, 71.4%; *p* < 0.0001) (Figure 6D).

## Discussion

In this study, we examined the correlation of the donor SMI and IMAC with graft survival and function in LDLT. A high donor IMAC was correlated with poor graft prognosis and graft function deterioration. We stratified LDLT patients by three risk factors, including high IMAC, high MELD score, and absence of splenectomy, and found that the presence of two or more risk factors significantly reduced graft survival.

The usefulness of the IMAC for predicting the prognosis of patients with various diseases, such as cirrhosis and pancreatic cancer, has been reported, and a high IMAC was shown to correlate with poor prognosis (26, 27). In LDLT, Hamaguchi et al. firstly reported a significant association between the recipient IMAC and recipient early mortality (8). Miyachi et al. reported that combined high SMI and IMAC in male donors was an independent protective factor against graft loss after LDLT (28). To our knowledge, this is the only report showing a correlation between donor muscle quality and quantity and graft mortality. Previous studies adjusted for SMI and IMAC with donor age because there was a strong correlation between donor age and donor SMI and IMAC. In our case, there was a significant, but not strong, correlation between the donor IMAC and age. The donor selection criteria varied among institutions, and we also used donor grafts from relatively elderly donors up to 65 years of age. Donors in their 50s and 60s are expected to be relatively healthy with good muscle quality and quantity. These differences in donor selection across facilities may have affected the relationship between donor age, IMAC, and SMI, and further investigations are needed in a larger cohort. Hence, we did not adjust the IMAC for donor age, and both the male and female donor IMAC showed a strong correlation with graft survival within 6 months. In our institution, donors whose body mass index is greater than 25 or who have fatty liver in the preoperative evaluation are placed on a diet before surgery. This may have influenced the relationship between donor age and preoperative IMAC and SMI.

In LDLT, there are rarely ideal conditions in terms of recipient status and donor selection. Some compromises are often necessary in donor selection because of organ shortages. Hence, we examined under which conditions IMAC assessment is useful. In low-risk recipients, whose MELD score was 20 or lower and who could not undergo splenectomy, there was a significant difference in 6-month graft survival rates between the high-IMAC group and low-IMAC group, albeit not by a large margin. Surprisingly, in the moderate-risk group, which included patients with one risk, i.e., high MELD score or absence of splenectomy, the difference was large and significant. This may indicate that donor selection considering the IMAC may be important for moderate-risk recipients scheduled to undergo LDLT. Splenectomy is a risk factor for SFSG syndrome in LDLT, and splenectomy is recommended for recipients with small grafts or portal hypertension (19). However, splenectomy is often difficult after partial splenic embolization or spontaneous bacterial peritonitis before transplantation. Our study showed that donor selection based on the IMAC may improve graft prognosis for recipients who cannot undergo simultaneous splenectomy. Although there have been several reports on graft quality assessment in LDLT, there has been no report on the effectiveness of quality assessment markers. In the future, qualitative markers that consider other background factors should be examined. Donor age has been used as a marker for graft quality in LDLT(18). However, liver graft quality does not uniformly decline with donor age, and it is important to assess individual changes in donor grafts because there are individual differences in aging (29). In addition, the several qualitative assessment methods previously reported require liver biopsy (30, 31), while this IMAC examination is not invasive, and the IMAC can be measured with CT images obtained before surgery, with no additional burden on the donor. In our study, the IMAC was a predictive factor for graft failure, although it was correlated with donor age. This suggests that the IMAC may represent individual biological aging rather than chronological aging, and further investigations are needed.

The relationships between the musculature and liver are not well understood. Interleukin-6 (IL-6), which is implicated in both liver regeneration and metabolic functions, is secreted into the bloodstream in response to muscle contraction (32). Some epidemiological studies have reported a negative association between the amount of regular body activity and resting plasma IL-6 concentrations(33). With exercise training, IL-6 downregulation is counteracted by increased IL-6 receptor (IL-6R) expression, resulting in increased sensitivity to IL-6 (32). We hypothesized that resting plasma IL-6 concentrations are upregulated by lack of muscle use in donors with a high IMAC, and IL-6R in the liver is downregulated, thereby decreasing sensitivity and disturbing hepatocyte regeneration.

The relationship between the IMAC and graft survival was more pronounced in women. The difference may result from expression of the estrogen receptor. Estrogen is one of the most important molecular markers of liver regeneration, and it has been reported that more estrogen receptors are expressed in the male liver than in the female liver(34,35). Hence, grafts from female donors may have more directly reflected the effects of IL-6.

This study has some limitations. First, this was a retrospective and single-center study. The IMAC needs to be studied on a large scale in the future, as it is a non-invasive examination and can be evaluated using CT scans performed preoperatively. Second, there is no clear answer as to whether the IMAC should be adjusted for age. Our study did not find a strong correlation with age, while others have reported strong correlations. In any case, the IMAC is a useful prognostic marker for graft survival in LDLT, but a large-scale validation may be needed in the future to determine which IMAC or adjusted IMAC is more useful. Third, there were some significant differences in patient characteristics between the high- and low-IMAC groups. We performed univariate and multivariate analyses to more accurately examine the correlation between the IMAC and patient characteristics (Supplementary Tables S1, S2) because there were several significant differences in patient characteristics between the high- and low-IMAC groups. A high MELD score was significantly correlated with a high IMAC in female donors. Conversely, there no parameters were significantly correlated with a high IMAC in male donors. In the future, we need to assess the patient characteristics of a different, larger cohort and re-examine the usefulness of the IMAC.

In conclusion, the donor IMAC correlates with graft survival. Thus, the donor IMAC may be useful for predicting graft function and, by extension, graft mortality.

## Data Availability

The raw data supporting the conclusion of this article will be made available by the authors, without undue reservation.

## Abbreviations

CT, computed tomography; DM, diabetes mellitus; GV/SLV, graft volume/recipient standard liver volume; HCC, hepatocellular carcinoma; HR, hazard ratio; ICU, intensive care unit; IL-6, interleukin-6; IMAC, high intramuscular adipose tissue content; LDLT, living donor liver transplantation; MELD, Model for End-Stage Liver Disease; POD, postoperative day; PT-INR, prothrombin-time international normalized ratio; SMI, skeletal muscle mass index; T-bil, total-bilirubin.
