# Supplementary material for: Donor Skeletal Muscle Quality Affects Graft Mortality After Living Donor Liver Transplantation- A Single Center, Retrospective Study
Source: Transpl Int. 2022 Dec 9;35:10723. doi: 10.3389/ti.2022.10723 (PMC9784912; doi:10.3389/ti.2022.10723)
Supplement: Supplementary file 1 [file DataSheet1.docx]

**Supplement Table 1. Univariate and multivariate analysis of high IMAC and clinicopathological factors in male donor**

|  | Univariate analysis | | | Multivariate analysis | | |
| --- | --- | --- | --- | --- | --- | --- |
| **Variables** | OR | 95%CI | P-value | OR | 95%CI | P-value |
| **Donor variables** |  |  |  |  |  |  |
| **Age (year)** | 1.02 | 0.99-1.04 | 0.1678 | 1.02 | 0.99-1.04 | 0.1099 |
| **Graft**  Right (n=90)  Others (n=145) | 1.00  1.75 | (reference)  1.02-2.98 | 0.0405 | 1.00  1.16 | (reference)  0.49-2.70 | 0.7391 |
| **GV/SLV (%)** | 0.97 | 0.94-0.99 | 0.0273 | 0.53 | 0.01-43.0 | 0.7770 |
| **GRWR (%)** | 0.26 | 1.04-3.80 | 0.0536 | 0.37 | 0.01-17.6 | 0.6162 |
| **ABO incompatible**  No (n=198)  Yes (n=37) | 1.00  1.30 | (reference)  0.64-2.63 | 0.4623 |  |  |  |
| **Recipient variables** |  |  |  |  |  |  |
| **Sex**  Male (n=93)  Female (n=142) | 0.65  1.00 | 0.38-1.09  (reference) | 0.1021 | 0.70  1.00 | 0.39-1.29  (reference) | 0.2555 |
| **Age (years)** | 1.01 | 0.98-1.03 | 0.6746 |  |  |  |
| **Hepatocellular disease**  No (n=75)  Yes (n=160) | 1.00  0.75 | (reference)  0.43-1.30 | 0.3117 |  |  |  |
| **HCC**  Without HCC (n=244)  With HCC (n=136) | 1.00  0.75 | (reference)  0.44-1.26 | 0.2777 |  |  |  |
| **Preoperative hospital treatment**  No (n=140)  Yes (n=95) | 1.00  1.05 | (reference)  0.62-1.81 | 0.8462 |  |  |  |
| **MELD score** | 1.00 | 0.96-1.04 | 0.8265 |  |  |  |
| **Splenectomy**  With splenectomy (n=195)  Without splenectomy (n=40) | 1.00  1.21 | (reference)  0.61-2.40 | 0.5975 |  |  |  |

GRWR, graft recipient weight ratio; GV/SLV, graft volume/recipient standard liver volume ratio; HCC, hepatocellular carcinoma; MELD, Model for End-Stage Liver Disease.

**Supplement Table 2. Univariate and multivariate analysis of high IMAC and clinicopathological factors in female donor**

|  | Univariate analysis | | | Multivariate analysis | | |
| --- | --- | --- | --- | --- | --- | --- |
| **Variables (N=145)** | OR | 95%CI | P-value | OR | 95%CI | P-value |
| **Donor variables** |  |  |  |  |  |  |
| **Age (year)** | 1.02 | 0.99-1.56 | 0.2254 |  |  |  |
| **Graft**  Right (n=106)  Others (n=39) | 1.00  1.06 | (reference)  0.49-2.31 | 0.8861 |  |  |  |
| **GV/SLV (%)** | 1.01 | 0.97-1.06 | 0.5276 |  |  |  |
| **GRWR (%)** | 1.59 | 0.20-12.5 | 0.6615 |  |  |  |
| **ABO incompatible**  No (n=119)  Yes (n=26) | 1.00  0.32 | (reference)  0.10-0.99 | 0.0488 | 1.00  0.37 | (reference)  0.12-1.16 | 0.0876 |
| **Recipient variables** |  |  |  |  |  |  |
| **Sex**  Male (n=72)  Female (n=73) | 1.09  1.00 | 0.54-2.18  (reference) | 0.8143 |  |  |  |
| **Age (years)** | 1.01 | 0.16-6.15 | 0.9949 |  |  |  |
| **Hepatocellular disease**  No (n=47)  Yes (n=98) | 1.00  1.62 | (reference)  0.75-3.52 | 0.2222 |  |  |  |
| **HCC**  Without HCC (n=104)  With HCC (n=41) | 1.00  1.51 | (reference)  0.71-3.21 | 0.2871 |  |  |  |
| **Preoperative hospital treatment**  No (n=99)  Yes (n=46) | 1.00  1.15 | (reference)  0.55-2.41 | 0.7071 |  |  |  |
| **MELD score** | 1.08 | 1.03-1.13 | 0.0007 | 1.08 | 1.03-1.13 | 0.0025 |
| **Splenectomy**  With splenectomy (n=126)  Without splenectomy (n=19) | 1.00  0.71 | (reference)  0.24-2.12 | 0.5438 |  |  |  |

GRWR, graft recipient weight ratio; GV/SLV, graft volume/recipient standard liver volume ratio; HCC, hepatocellular carcinoma; MELD, Model for End-Stage Liver Disease.
